# Social demographics determinants for resistome and microbiome variation of a multiethnic community in Southern Malaysia

**DOI:** 10.1038/s41522-023-00425-0

**Published:** 2023-08-12

**Authors:** J. Dwiyanto, M. A. L. Huët, M. H. Hussain, T. T. Su, J. B. L. Tan, K. Y. Toh, J. W. J. Lee, S. Rahman, C. W. Chong

**Affiliations:** 1 AMILI, Singapore, 118261 Singapore; 2 Department of Medical Microbiology, Faculty of Medicine, University of Malaya, Kuala Lumpur, 50603 Malaysia; 3 Faculty of Science, University of Mauritius, Reduit, 80837 Mauritius; 4 School of Science, Monash University Malaysia, Bandar Sunway, 47500 Malaysia; 5 South East Asia Community Observatory, Segamat, 85000 Malaysia; 6 Department of Medicine, National University Hospital, Singapore, 119228 Singapore; 7 Tropical Medicine and Biology Platform, Monash University Malaysia, Bandar Sunway, 47500 Malaysia; 8 School of Pharmacy, Monash University Malaysia, Bandar Sunway, 47500 Malaysia

**Keywords:** Antimicrobials, Microbiome, Microbial genetics

## Abstract

The prevalence of antibiotic-resistant bacteria in Southeast Asia is a significant concern, yet there is limited research on the gut resistome and its correlation with lifestyle and environmental factors in the region. This study aimed to profile the gut resistome of 200 individuals in Malaysia using shotgun metagenomic sequencing and investigate its association with questionnaire data comprising demographic and lifestyle variables. A total of 1038 antibiotic resistance genes from 26 classes were detected with a mean carriage rate of 1.74 ± 1.18 gene copies per cell per person. Correlation analysis identified 14 environmental factors, including hygiene habits, health parameters, and intestinal colonization, that were significantly associated with the resistome (adjusted multivariate PERMANOVA, *p* < 0.05). Notably, individuals with positive yeast cultures exhibited a reduced copy number of 15 antibiotic resistance genes. Network analysis highlighted *Escherichia coli* as a major resistome network hub, with a positive correlation to 36 antibiotic-resistance genes. Our findings suggest that *E. coli* may play a pivotal role in shaping the resistome dynamics in Segamat, Malaysia, and its abundance is strongly associated with the community’s health and lifestyle habits. Furthermore, the presence of yeast appears to be associated with the suppression of antibiotic-resistance genes.

## Introduction

Antimicrobial resistance poses a significant global health threat, projected to result in an annual financial burden of USD 1 trillion and a global mortality rate of 10 million by 2050^1^. While concerns about antibiotic resistance have been raised since the early days of antibiotic discovery in the 1940s^2^, the crucial role of antibiotics in clinical therapeutics and the animal food industry has led to their increased usage over time^3^, resulting in a surge of antibiotic resistance worldwide. Unfortunately, the traditional approacssh of discovering novel antimicrobials to combat resistance is unsustainable, as the rate of such discoveries has significantly slowed down^4^. Urgent efforts are required to halt the rapid emergence of resistance.

A comprehensive antibiotic stewardship program is widely considered as the most effective solution to manage the occurrence of antibiotic resistance in a country or region. Central to an effective antibiotic stewardship policy is regular antibiotic surveillance^5^. While antibiotic surveillance is commonly practised in clinical settings, it is seldom utilized in non-clinical settings^6^, such as at the community level, despite the increasing trend of community-associated resistant infections, including MRSA and ESBL^7,8^. The lack of antibiotic surveillance at the community level is especially true in low and middle-income countries, where antibiotic resistance is highly prevalent^9^.

Southeast Asia is recognized as a global hotspot for antibiotic resistance. Multiple studies have highlighted the risk of acquiring antibiotic-resistance genes (ARGs) following travel to the region^10,11^. Furthermore, the Southeast Asian communities exhibit some of the highest carriage burdens of antibiotic resistance genes. For instance, a Thai community reported a 72.6% faecal colonisation rate with ESBL-producing Enterobacteriaceae, one of the highest rates ever recorded^12^. This finding was supported by a recent meta-analysis, which identified South East Asia as the region with the highest carriage rate of ESBL-producing *Escherichia coli* among healthy community members^13^. Despite the high burden of antibiotic resistance in the region, comprehensive resistome profiling studies are severely lacking. Among the limited studies available, Pereira-Dias et al. (2021)^14^ discovered a wide array of antibiotic-resistance genes in a cohort of 42 healthy Vietnamese individuals. However, the study was limited in scale and lacked complementary environmental and lifestyle data, hampering its interpretation. More recently, Li et al. (2022) reported the resistome profiles of Singaporeans through urban sewage surveillance^15^. While wastewater surveillance is a practical method^16^, its findings are highly dependent on the country’s wastewater treatment system^17^, which may underestimate the actual resistome burden in an area. In Malaysia, metagenomic-based resistome studies are limited in size and scale and are not specifically designed for surveillance purposes^17,18^. Additionally, much of our current knowledge is based on culture-dependent resistance studies (e.g.^19,20^), which have inherent limitations and cannot provide a comprehensive overview of a community’s resistome profile. This knowledge gap hinders our understanding of the dynamics of antibiotic resistance in the region, their interaction with the host’s intestinal environment, and the risk factors associated with acquiring the diverse array of ARGs within the community.

Both hygiene practices and exposure to antibiotics have been identified as significant factors influencing the development and spread of antibiotic resistance. Poor hygiene practices can increase exposure to bacteria carrying ARG and facilitate the transfer of resistance genes between bacteria through horizontal gene transfer. Indeed, recent findings by Ramay et al. (2020)^21^ indicate that households in Guatemala with better hygiene practices have a lower likelihood of being colonized by antibiotic-resistant bacteria. Supporting this observation, a nationwide action plan involving 685 South African households also confirmed the importance of hygiene factors in controlling antimicrobial resistance prevalence, reducing infection rates, and subsequently minimizing the administration of antimicrobials^22^. It is widely accepted that the prescription of antimicrobials directly contributes to the selection pressure of antibiotic-resistant organisms^23^. Given these facts, it is crucial to investigate whether hygiene-related habits practised by the Southeast Asian community contribute to the endemicity of antibiotic resistance in the region, which we hypothesized play an important role in the dynamic of ARG in the community. Such investigations can inform policymakers about effective action plans required to tackle this issue.

In our previous research, we examined the 16 S rRNA-based gut microbial composition of community dwellers in Segamat, a district in Southern Malaysia^24^, and profiled the colonisation burden of ESBL-producing *E. coli* using whole-genome sequencing^25^. This study expands upon our previous findings by utilizing shotgun metagenomic sequencing to relate the resistome profiles of community members to their microbiota and lifestyle behaviours.

## Results

### Cohort overview

A total of 200 community participants from 102 households were recruited from the South East Asia Community Observatory (SEACO) cohort from May through June 2018 (Table 1, Supplementary Table 1)^26^. The cohort consisted of approximately equal numbers of Chinese (*n* = 64), Jakun (*n* = 46), Indian (*n* = 45), and Malays (*n* = 45). The participants were reasonably balanced in terms of sex distribution, with 107 females and 93 males. The age distribution of the participants was similar to that of the SEACO cohort, with a slightly higher frequency of participants aged 11–20. Most households earned between MYR1,000-MYR5,000 monthly (Supplementary Fig. 1). Females predominantly identified as homemakers (*n* = 56/107), while males primarily worked in the agriculture sector (*n* = 36/93) (Supplementary Fig. 2).

**Table 1:** Demographic distribution of the studied Segamat cohort with *P* value obtained through chi-square analysis.

	Chinese	Indian	Jakun	Malay	*P* value
Female	36 (56%)	22 (49%)	28 (61%)	21 (47%)	0.49
Age	50 ( ± 21)	49 ( ± 16)	35 ( ± 18)	41 ( ± 21)	0.0004
Age (Decade)					0.005
<11	0 (0%)	0 (0%)	2 (4%)	2 (4%)	
11–20	11 (17%)	4 (9%)	13 (28%)	10 (22%)	
21–30	3 (5%)	2 (4%)	5 (11%)	2 (4%)	
31–40	0 (0%)	6 (13%)	7 (15%)	6 (13%)	
41–50	11 (17%)	14 (31%)	9 (20%)	5 (11%)	
51–60	18 (28%)	8 (18%)	6 (13%)	10 (22%)	
61–70	14 (22%)	7 (16%)	3 (7%)	9 (20%)	
71–80	4 (6%)	3 (7%)	1 (2%)	1 (2%)	
81–90	3 (5%)	1 (2%)	0 (0%)	0 (0%)	
>90	0 (0%)	0 (0%)	0 (0%)	0 (0%)	
Subdistrict					<0.0001
Bekok	64 (100%)	0 (0%)	46 (100%)	0 (0%)	
Chaah	0 (0%)	45 (100%)	0 (0%)	0 (0%)	
Jabi	0 (0%)	0 (0%)	0 (0%)	45 (100%)	
Education					0.0003
Degree	3 (5%)	2 (4%)	0 (0%)	0 (0%)	
Diploma	2 (3%)	2 (4%)	0 (0%)	1 (2%)	
DNC_primary^a^	18 (28%)	8 (18%)	16 (35%)	9 (20%)	
No	1 (2%)	1 (2%)	11 (24%)	1 (2%)	
PMR^b^	15 (23%)	11 (24%)	8 (17%)	6 (13%)	
Primary	15 (23%)	11 (24%)	9 (20%)	16 (36%)	
SPM^c^	9 (14%)	10 (22%)	1 (2%)	12 (27%)	
Missing	1 (2%)	0 (0%)	1 (2%)	0 (0%)	
Occupation					0.0003
Agricultural	17 (27%)	4 (9%)	11 (24%)	9 (20%)	
Children	11 (17%)	1 (2%)	6 (13%)	11 (24%)	
Craft	0 (0%)	2 (4%)	1 (2%)	1 (2%)	
Elementary	0 (0%)	2 (4%)	0 (0%)	1 (2%)	
Homemaker	13 (20%)	14 (31%)	17 (37%)	12 (27%)	
Operator	1 (2%)	2 (4%)	0 (0%)	0 (0%)	
Others	0 (0%)	2 (4%)	0 (0%)	0 (0%)	
Professional	1 (2%)	1 (2%)	0 (0%)	0 (0%)	
Self-employed	3 (5%)	1 (2%)	1 (2%)	3 (7%)	
Service	4 (6%)	6 (13%)	0 (0%)	7 (16%)	
Technician	0 (0%)	0 (0%)	1 (2%)	0 (0%)	
Unemployed	13 (20%)	10 (22%)	9 (20%)	1 (2%)	
Missing	1 (2%)	0 (0%)	0 (0%)	0 (0%)	
Blood Pressure					0.86
Elevated	12 (19%)	9 (20%)	7 (15%)	6 (13%)	
Normal	23 (36%)	14 (31%)	13 (28%)	12 (27%)	
Stage1	15 (23%)	13 (29%)	19 (41%)	15 (33%)	
Stage2	6 (9%)	6 (13%)	5 (11%)	3 (7%)	
Missing	8 (12%)	3 (7%)	2 (4%)	9 (20%)	
Body Mass Index					0.004
Normal	39 (61%)	13 (29%)	19 (41%)	18 (40%)	
Obese	6 (9%)	15 (33%)	10 (22%)	10 (22%)	
Overweight	12 (19%)	16 (36%)	9 (20%)	9 (20%)	
Underweight	6 (9%)	1 (2%)	8 (17%)	6 (13%)	
Missing	1 (2%)	0 (0%)	0 (0%)	2 (4%)	
Income					<0.0001
<MYR400	1 (2%)	0 (0%)	2 (4%)	0 (0%)	
MYR401-700	4 (6%)	9 (20%)	11 (24%)	2 (4%)	
MYR701-1000	11 (17%)	3 (7%)	3 (7%)	0 (0%)	
MYR1001-5000	41 (64%)	29 (64%)	29 (63%)	43 (96%)	
>MYR5000	7 (11%)	4 (9%)	1 (2%)	0 (0%)	

^a^DNC = Did Not Complete.

^b^PMR = Penilaian Menengah Rendah/Form Three Secondary Assessment.

^c^SPM = Sijil Pelajaran Malaysia/Form Five Secondary Assessment/O level equivalent.

Of the participants, 65.5% (*n* = 131/200) reported not suffering from any chronic diseases. Among those who did report chronic diseases (*n* = 69/200), hypertension was the most frequently reported condition (*n* = 43/69), followed by high blood cholesterol (*n* = 25/69) and diabetes (*n* = 23/69). The participants reported active consumption of drugs related to non-communicable diseases, with amlodipine being the most frequently reported (*n* = 15), followed by simvastatin (*n* = 14) and metformin (*n* = 12). None of the participants reported being on antibiotics at the time of sampling.

### Resistome profile of the Segamat cohort

**Fig. 1:**
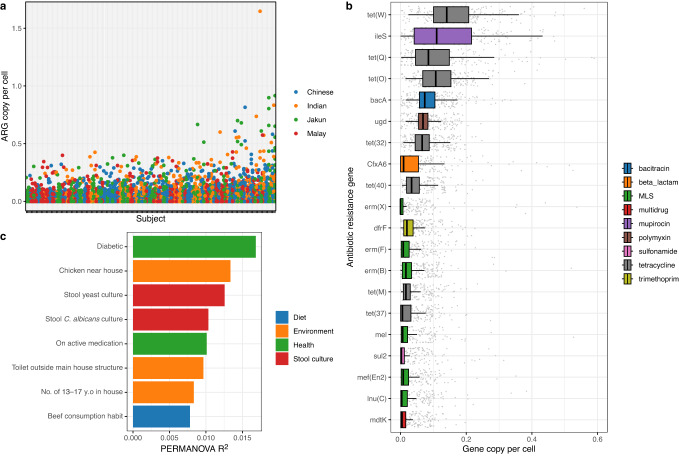
The abundance and distribution of antibiotic resistance genes detected from the Segamat cohort along with their associated lifestyle factors. **a** Distribution of antibiotic resistance genes across subjects (*N* = 200); (**b**) Top 20 antibiotic resistance genes detected in the Segamat cohort based on gene copies per cell; (**c**) Factors associated with the resistome abundance profile based on PERMANOVA adjusted for batch, household, age, sex, ethnicity, income, and BMI (*p* < 0.05). The boxplot’s lower and upper boundaries marked the first (25th percentile) and the third (75th percentile) quartile of the visualised data, while the middle hinge marked the median. The lower and upper whiskers marked the lowest and highest values no smaller/larger than 1.5× interquartile range of the visualised dataset, respectively.

A total of 1038 resistance genes were identified from the 200 community participants, conferring resistance to 26 classes of ARG (Fig. 1a). The mean number of resistance genes carried per person was 1.74 ± 1.18 gene copies per cell. After filtering out low-abundance (<0.5%) and non-prevalent (<10%) genes, 66 resistance genes remained, conferring resistance to 14 types of ARG (Supplementary Table 2). The tetracycline resistance genes were the most abundant (mean 0.63 ± 0.28 gene copy per cell), followed by macrolide-lincosamide-streptogramine (MLS) (0.19 ± 0.23), mupirocin (0.13 ± 0.11), multidrug resistance genes (mainly *emr* [*n* = 4/14] and *mdt* [*n* = 5/14] efflux pumps) (0.13 ± 0.22), and polymyxin (0.11 ± 0.09). Individually, *tetW* was the most abundant (0.17 ± 0.09), followed by Bifidobacteria-specific *ileS* (0.13 ± 0.11), tetQ (0.13 ± 0.14), *tetO* (0.12 ± 0.08), and *bacA* (0.09 ± 0.05). Three *Escherichia coli*-specific resistance genes were also detected, namely the multidrug resistance gene *mdfA* (0.01 ± 0.02) and *emrE* (0.01 ± 0.02), and the *ampC* ß-lactamase (0.005 ± 0.01).

### Shannon diversity of resistance genes across demographic factors

No demographic factors were found to be associated with the resistome Shannon diversity (linear mixed model adjusted for batch and household as random effects, and age, sex, ethnicity, gender, and BMI as fixed effect, LRT *p* > 0.1). When tested across 56 lifestyle, hygiene, and health parameters, eight factors exhibited significant association with the resistome Shannon diversity (linear mixed model LRT *p* < 0.1, Supplementary Fig. 3, Supplementary Table 3). Notably, resistome diversity was negatively associated with positive laboratory culture of mould and yeast in the participants’ stool samples and the number of household members. Conversely, it was positively associated with medical conditions (being on active medication and suffering from diabetes).

### ß-diversity analysis of the resistome profile

A ß-diversity analysis was conducted to compare the compositional differences of the resistome profile across demographic and lifestyle variables. PERMANOVA analyses on the Aitchison’s distance were performed, controlled for batch and household variations. Subgroup analyses were conducted to assess whether the factors identified through univariate PERMANOVA analyses were universal across demographic parameters (i.e., age, sex, ethnicity, income, BMI). The resistome profile of the different subgroup of each demographic section seemed to be affected by different lifestyle variables, which comprised gut colonisation, dietary, health and hygiene-related parameters (Supplementary Fig. 4). To account for this variation, we conducted PERMANOVA analysis adjusted for these demographic variables (i.e., age, sex, ethnicity, income, BMI) along with batch and household. As a result, eight factors were identified as significantly associated with the resistome profile (PERMANOVA *p* < 0.05, Fig. 1c, Supplementary Table 4). These variables overlapped with those found to be associated with the resistome Shannon diversity, including gut colonization (positive culture of yeast and *C. albicans*), hygiene-related parameters (toilet location), and health parameters (active medication status and suffering from diabetes). Additionally, diet (beef consumption habit) and environmental factors (chicken in the house vicinity, number of people in the house) were also linked to the resistome profile.

**Fig. 2:**
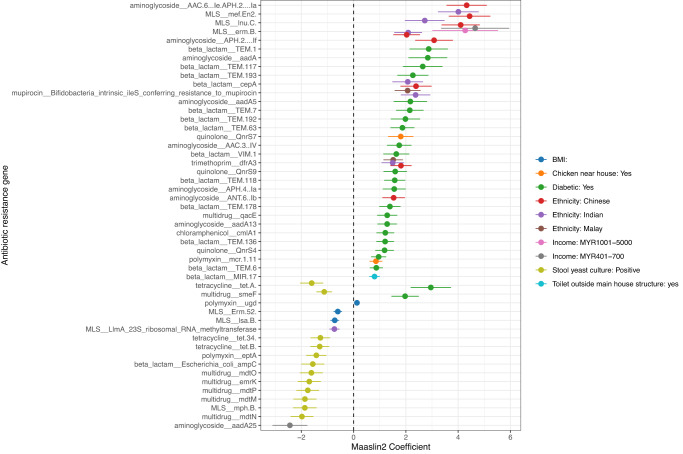
Differentially abundant antibiotic resistance genes against lifestyle and demographic factors of the Segamat cohort, analysed using Maaslin2 (*q* < 0.1). Ethnicity and household income variables were compared against Jakun and those earning <MYR400, respectively. Error bars are visualised based on the standard errors as reported in the Maaslin2 output.

Multivariate Maaslin2 differential abundance analysis, based on these eight variables and adjusted for batch and household clustering as random effects and age, sex, ethnicity, income, and BMI as fixed effects, revealed significant associations of 49 ARGs with seven lifestyle and demographic variables (Maaslin2 linear model, log-transformed, *q* < 0.1, Supplementary Table 5, Fig. 2). Notably, suffering from diabetes was positively associated with 23 ARGs, while a positive yeast culture was linked to a reduced copy number of 12 genes.

### Microbial diversity and community composition associates with demographic and lifestyle factors

A total of 685 gut microbial species were detected, with 196 species retained after excluding those with low abundance and prevalence. Bacteria constituted the majority of the reads (mean abundance 96.99 ± 4.29%), followed by archaea (0.51 ± 1.85%) and viruses (0.07 ± 0.36%). Among the bacteria kingdom, Firmicutes had the highest mean abundance (38.96 ± 12.87%), followed by Actinobacteria (36.52 ± 19.45%), Bacteroidetes (17.58 ± 13.90%), and Proteobacteria (3.17 ± 5.38%), with other phyla exhibiting <1% mean relative abundance. A total of 83 genera were identified, with *Bifidobacterium* being the most abundant, followed by *Collinsella*, *Prevotella*, *Bacteroides*, *Blautia*, and *Faecalibacterium*, each with >5% abundance (Supplementary Fig. 5). *Bifidobacterium adolescentis*, *Collinsella aerofaciens*, *Prevotella copri*, and *Bifidobacterium longum* were the most prevalent species. *E. coli* was the only Proteobacteria detected among the top 20 most abundant species.

None of the demographic factors were significantly associated with the Shannon diversity of the species profile (Linear mixed model, adjusted for batch and household as random effects, and age, sex, ethnicity, BMI, and income as fixed effects, LRT *p* > 0.1). Among the lifestyle variables tested, exercise frequency, fermented food consumption frequency, and access to piped drinking water were all found to be associated with the microbiota Shannon diversity (linear mixed model, LRT *p* < 0.1, Supplementary Table 6, Supplementary Fig. 6).

Seven lifestyle variables were associated with the gut microbial composition (PERMANOVA adjusted for age, sex, ethnicity, income, BMI, batch, and household, *p* < 0.05, Supplementary Table 7). Some of these variables were similar to those associated with the resistome profile, comprising health parameters (suffering from diabetes and other diseases, surgical history), environmental (number of children aged six and below in the household, and total number of household members), and hygiene (having flush toilet type).

**Fig. 3:**
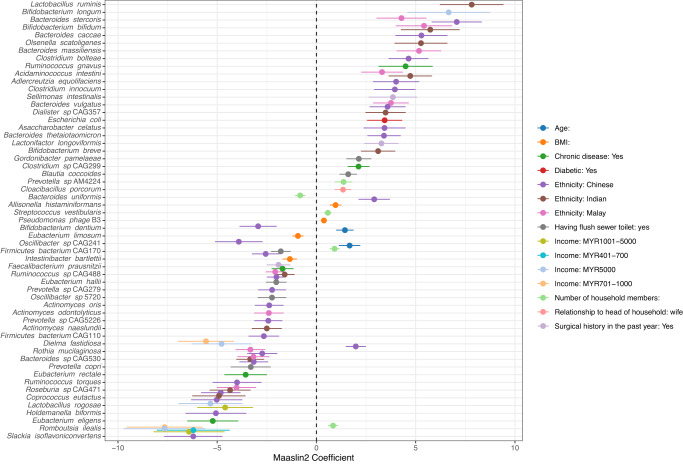
Differentially abundant species against lifestyle and demographic factors of the Segamat cohort, analysed using Maaslin2 (q < 0.1). Ethnicity, and household income variables were compared against Jakun and those earning <MYR400, respectively. Error bars are visualised based on the standard errors as reported in the Maaslin2 output.

Multivariate analysis of these lifestyle variables, adjusted for batch and household clustering as random effects and age, sex, ethnicity, income and BMI as fixed effect, detected 57 differentially abundant species across ten factors (Maaslin2, *q* < 0.1, Fig. 3). Ethnicity represented most of the observed variation (*n* = 46/57), followed by income (*n* = 9), toilet type (*n* = 6), and number of household members (*n* = 5). Notably, *E. coli* was positively associated with diabetes.

### Correlation of resistome profile with species profiles

**Fig. 4:**
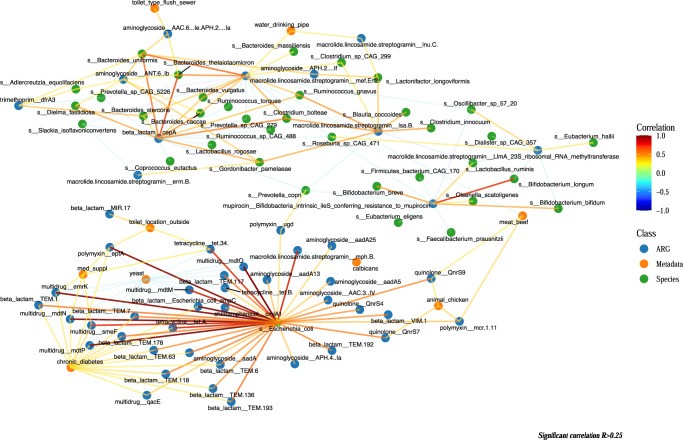
Network of demographic, lifestyle and environmental variables, species, and resistome features obtained from the Segamat community. Only significant (*p* < 0.05) Spearman correlation with absolute correlation >0.25 were shown.

A network analysis was conducted to examine the correlations between resistome features, microbial species, and metadata, which included 57 species, 49 ARGs, and 15 metadata features. The visualized network revealed negative associations between yeast and fifteen ARGs, as well as a positive association between diabetic patients and multiple ARGs (Fig. 4). Additionally, a link was observed between the presence of chickens near the house and two quinolone genes (*qnrS9* and *qnrS7*). Consumption history of beef meat was also linked to *qnrS9*. Most crucially, the network analysis highlighted the strong role of *E. coli* on the resistome profile of the Segamat cohort, being positively correlated with 36 ARGs. Furthermore, there was also a strong association between *E. coli* and the Shannon resistome diversity (Supplementary Fig. 7, linear model *p* < 0.05), further consolidating the observed association between *E. coli* and antibiotic resistance in Segamat.

## Discussion

In this report, we characterized the gut resistome profile of community dwellers through shotgun metagenomic sequencing in Malaysia. We did not observe any specific demographic factor with links to the gut resistome profile, suggesting that ARG was highly prevalent in the community regardless of age, sex, or ethnicity, confirming the endemicity of antibiotic resistance in Segamat. We discovered a strong relationship between the gut resistome profile with lifestyle and environmental factors related to hygiene, health, the environment, and dietary habits. Importantly, the resistome profile also seemed to be strongly linked to the abundance of *E. coli* and positive yeast intestinal colonisation.

Humans can be exposed to antibiotics through various routes, including medical prescriptions^27,28^, over-the-counter medications^29,30^, and the consumption of food products containing antibiotic residues^31–33^. Once ingested, antibiotics exert a direct selection pressure on the bacterial population, which could lead to the emergence of antibiotic-resistant strains through mutations and their dissemination via mobile genetic elements^34,35^. Medical antibiotic prescriptions typically ensure optimal dosage is prescribed to minimize the risk of antibiotic resistance development^36^. However, the availability of antibiotics without prescriptions in large parts of Southeast Asia^37,38^ has exposed the community to inappropriate and possible sub-dosage antibiotic usage, which could hasten the development of antimicrobial resistance to sublethal doses^39,40^ and potentially lead to high-level resistant strains.

Antibiotic consumption frequency varies widely by country and region and is linked to the effective implementation of proper antibiotic stewardship programs^41^. Although Malaysia is one of the few countries in Southeast Asia that has established a national antibiotic surveillance system and stewardship program under World Health Organization methodologies^42^, official antibiotic consumption data in the country is likely to underreport the true consumption rate due to a lack of reporting from the private sectors^42^. This issue is compounded by the fact that antibiotics are frequently available without proper prescription^38^. Nevertheless, Browne et al. (2021)^43^ performed spatial modelling of antibiotic usage in 204 countries through a compilation of surveys and official data sources up to 2018, finding Southeast Asia to be one of the largest consumers of global antibiotics, responsible for 6.5% (95% CI 9.4 – 12.0) of global antibiotic consumption. The high consumption frequency reported is aligned with a 2019 report suggesting the endemicity of antibiotic resistance in Southeast Asia^44^. Peering deeper into the region, Pereira-Dias et al. (2021)^14^ recently reported the gut resistome of 42 children and healthy adults from Vietnam, detecting 132 ARGs, which is much lower than the 1038 ARGs detected in this cohort. However, our study also utilized a much larger sample size (*n* = 200 versus *n* = 42) and sequencing reads (mean ~26 million reads per subject versus 12 million reads). Additionally, the distinct gene abundance also likely arose from the different reference databases utilized, where Pereira-Dias et al.^14^ used the SRST2 database compared to the CARD and ARDB database used by the ARGs-OPA pipeline in this study. Nevertheless, our results are more or less comparable with the reported 764 gene ARG subtypes from neighbouring Singapore, which also observed the dominance of tetracycline and aminoglycoside resistance genes in urban wastewater^15^. Regardless of the observed quantity, the high diversity of ARGs observed in our cohort is aligned with other studies indicating the endemicity of antibiotic resistance in Southeast Asia^14,45^. In particular, the high prevalence of resistance genes against aminoglycosides, cephalosporins, macrolides, and fluoroquinolones is a significant concern, considering their status as critically important antimicrobials for the treatment of infectious diseases^46^.

Agriculture is the main occupation of the subjects studied in this cohort, where tetracycline usage is relatively common^47–50^. The high abundance of tetracycline resistance observed in this study could therefore be a manifestation of direct occupational exposure, which has been reported^51^.

Hygiene factors are known to play an essential role in shaping the dynamics of antibiotic resistance. Poor hygiene practices such as inadequate handwashing^52^ or consumption of contaminated food and water^53^ can expose humans to bacteria that possess ARGs. Moreover, poor hygiene practices in healthcare settings have also been linked to the dissemination of antibiotic-resistant organisms^54^. Such exposure could facilitate the dissemination of ARG through horizontal gene transfer of mobile genetic elements such as plasmids, leading to the colonization of commensal bacteria carrying ARGs. In this study, hygiene factors such as water sources as well as the type and location of the toilet correlated significantly with the observed resistome. Antibiotic residues and resistant strains have been reported from different drinking water sources^55–57^, including in Malaysia^58^. Our finding thus suggests the possibility of exposure through the environment. This was also supported by the lower resistome Shannon diversity of subjects who had access to piped water, suggesting that untreated water source might play an important role in the transmission dynamic of ARG in the region.

Recent studies have revealed a correlation between body mass index (BMI) and gut microbiome diversity, with obese individuals exhibiting a lower gut microbial diversity than lean individuals^59,60^. The mechanism behind this correlation is likely driven by the dietary habits of individuals. Obese individuals tend to consume a diet low in plant-based foods^61^, which is a rich source of short-chain fatty acids^62^ and has been associated with a higher diversity of microbial species in the gut^63,64^. Additionally, the typical Western diet rich in fat and simple sugar, which has been associated with lower microbiome diversity, is often associated with gut inflammation^65,66^.

Southeast Asia is infamous for its high antibiotic usage owing to the lack of antibiotic regulation enforcement, with Malaysia recording one of the highest antibiotic use rates in the world^43^. Indeed, a high proportion of Malaysians have reported self-medication with antibiotics without prescriptions^67^ due to low awareness of antibiotic resistance^68^, compounding the issues of excessive and inappropriate antibiotic prescription in the country^69^. It is not surprising that subjects with health issues in this cohort were also associated with higher resistome abundance.

Opportunistic pathogens such as *E. coli* have been associated with bacterial infections (e.g., gastroenteritis^70^, urinary tract infection^71^, inflammatory bowel disease^72^) and non-communicable diseases (e.g., type II diabetes^73^ and hypertension^70^). In our previous study, we reported on the frequent carriage of multiple plasmid groups among ESBL-producing *E. coli*, with a phenotypic multidrug resistance rate of 90.29% in Segamat^25^. The observed correlation between *E. coli* abundance and resistome diversity reported in this study therefore corroborated our previous finding. The high prevalence of *E. coli* in diabetic subjects might explain the strong correlation between the disease and the diversity of the resistome. Such observation is perhaps unsurprising, given that diabetic individuals have been reported to have elevated *E. coli* abundance^73,74^ and are more susceptible to *E. coli*-associated antibiotic-resistant infections^75–77^. Most importantly, although 95% of the subjects carried *E. coli*, subjects with low *E. coli* abundance (i.e., lower than the population median of 0.32%) exhibited no significant association between species and resistome diversity, strongly suggesting the vital role *E. coli* plays in the resistome profile of the Segamat cohort.

Competition for space in the gut environment has been associated with health effects. For example, individuals infected with soil-transmitted helminths exhibit higher gut diversity^78^, commonly regarded as a health indicator^79^. Additionally, yeasts such as *C. albicans* have been reported to have an antagonistic relationship with gut bacteria^80^. The negative association between yeast colonization and numerous antibiotic genes observed might indicate the suppression of bacterial strains carrying ARGs. For example, yeast exhibits antibacterial activity against *E. coli*^81^, although the opposite has also been reported^82^. Regardless, our observation provides a point for further study that could potentially unveil the role of gut eukaryotes in the fight against antibiotic resistance.

Although antibiotic resistance is generally associated with bacteria, an increasing number of studies have reported the emergence of yeasts and fungi harbouring ARGs (for example^83^, and^84^). Crucially, horizontal gene transfer of resistance genes has been reported in yeasts both in controlled laboratory settings^85^ and from environmental data^86–88^ and community observations. Unlike in prokaryotes, the mechanisms of horizontal gene transfer among fungal and yeast cells are not well understood^88,89^.

In addition to the above factors, diet and economic status were also significantly associated with the resistome in this cohort. Diet has been reported as a major driver of the gut microbiota^90^. The positive association between beef consumption and quinolone resistance that we observed has also been reported^91,92^. Meanwhile, a higher prevalence of antibiotic resistance in lower-income individuals has also been reported^93^.

The strength of this study lies in its ability to link the gut resistome with both the gut microbiota and lifestyle and environmental data obtained through the administration of a comprehensive questionnaire, providing insights into the profile and potential acquisition pathways of ARGs in Segamat. However, our data alone was insufficient to elucidate the relationship of the observed gut resistome profile with other settings such as the animal, environmental, and the clinics. Such information is crucial to establish a clear link between the observed community profile and the other settings, establishing a One Health outlook on the resistome dynamics in the region. Additionally, the lack of stool consistency data from the participants, which could provide an indication of gastrointestinal health, might introduce some bias to our findings^94,95^. However, questions on long-term gastrointestinal complaints were part of the questionnaire, with none of the participants reporting any current or long-term gastrointestinal complaints. Although none of the participants were under antibiotic medications during the time of sampling, the recent history of antibiotic consumption was not known, which might inflate the abundance of ARG profiles for some subjects. The lack of long-read sequencing also limited plasmid assembly, which could inform the proportion and transmission capability of plasmid-mediated genes, is also missed. Lastly, the cross-sectional nature of this study prevents insights into the stability and transmission patterns of the observed resistome profiles of the community members. Regardless, this study confirms the presence of massive antibiotic resistance reservoirs among the community dwellers in Malaysia and unveils the link between the observed resistome and lifestyle and demographic factors, providing crucial insights to help develop proper interventional and stewardship policies in the region.

## Methods

### Recruitment

This study has been approved by the Monash University Human Research Ethics Committee (MUHREC, project no. 1516) and adheres to the Declaration of Helsinki. This study was conducted in Segamat, a district located in the southern state of Johor, Malaysia. The recruitment procedure has been reported^24^. Briefly, Segamat community dwellers under the South East Asia Community Observatory (SEACO)^26^ community cohort were recruited from May through June 2018. Each participant provided written informed consent, and parental/guardian written consent was obtained for individuals <18 years of age.

### Sample collection

The data collection procedure used in this study has been reported^24^. Briefly, information on the demographics and lifestyles habits of the participants were obtained through a face-to-face interview with trained SEACO data collectors. Afterwards, the participants were provided with a styrofoam box filled with ice packs and a Fisherbrand™ Commode Specimen Collection Kit (Fisher Scientific) and briefed on the sample collection procedure. The stool samples were then pooled and transported to Monash University Malaysia within 24 h and stored at −50 °C until further processing.

### Identification of positive yeast colonisation

The procedure used to screen for yeast, mould, and ESBL has been published^96^. Briefly, a fraction of the fresh stool specimens was enriched in buffered peptone water in a 1:9 (w/v) ratio and then cultured on their respective screening media through the spread plate technique. The enriched specimen was then cultured on potato dextrose agar and incubated overnight at 25 °C. Colonies with yeast-like morphology were confirmed through biochemical and sequencing.

### DNA extraction and metagenomic sequencing

The faecal samples were mixed with Zymo Research DNA/RNA Shield™ in a 1:9 (w/v) ratio. The DNA was extracted using the QIAamp PowerFecal Pro DNA Kit (Qiagen) as per the manufacturer’s protocol. Afterwards, the extracted DNA was sent to Macrogen Singapore (Macrogen Asia Pacific Pte Ltd, Singapore) for library preparation and shotgun sequencing using a 2 × 250 bp configuration in an Illuemina NovaSeq sequencing platform.

### Annotation of species, pathway, and antibiotic resistance genes composition

The resulting raw reads (total 5,400,890,140 reads, mean 26,345,806 ± 5,676,204 reads per sample) were then imported to BioBakery3 workflows version 3.0.0-alpha.6^97^. Briefly, BioBakery3 workflows is an assembly of programmes to process shotgun metagenomic sequencing data from start to finish^97^. As part of the workflow, removal of adapters and sequencing primers and decontamination of human reads were conducted using KneadDaa version 0.7.6. The total reads after decontamination using KneadData was 4,736,421,077, with mean of 23,104,493 ± 5,219,100. The inference of species abundance was conducted using MetaPhlAn3 version 3.0.1 with sequence aligned using bowtie2 version 2.2.3.

ARGs abundance was annotated from the decontaminated sequence files with the Online Analysis Pipeline for Antibiotic Resistance Genes Detection from Metagenomic Data Using an Integrated Structured ARG Database (ARGs-OAP) pipeline version 3.2.2^98^. ARGs-OAP utilised a structured ARG reference database, which integrated both ARDB and CARD and provides a cell number-normalised resistance gene reading. The database is regularly updated with their Linux implementation available at https://github.com/xinehc/args_oap.

### Data analysis

Data analysis was conducted in R version 4.1.2^99^ under the tidyverse version 1.3.1 environment^100^. Demographic statistics description was tabulated using the R package Gmisc version 2.1.0^101^. The microbiome data was then compiled into phyloseq objects using the R package phyloseq version 1.38.0^102^. The same package was used to estimate the Shannon diversity scores of the samples with the function estimate_richness, a wrapper function from the vegan package version 2.5–7^103^.

Alpha-diversity analysis for numeric variables was conducted using linear mixed model under the R package lme4 version 1–1.29 to account for the nested design of the subjects by household. Likelihood ratio test was conducted to determine significance. Non-numerical variables were analysed using Kruskal-Wallis test, and pairwise Wilcoxon test with Benjamini-Hochberg correction was used for post-hoc comparison. Before ß-diversity analysis, the microbiome profiles were filtered to remove features with <0.5% abundance and <10% prevalence using the core function of the microbiome package version 1.16.0^104^. This filtration criterion retained 66 of 1038 resistance genes and 196 of the 685 species detected in the species composition profile. The abundance data were centred log ratio-transformed using the propr function in the homonymously named R package version 4.2.6^105^. Permutational multivariate analysis of variance (PERMANOVA) was performed to identify variables significantly associated with microbiome profiles using the function adonis from the R package vegan version 2.5–7^103^. Significantly different species and ARG across variables were identified using the R package Maaslin2 version 1.8.0^106^, using linear model. All plots were visualised using the R package ggplot2 version 2_3.3.5^107^. Correlation analysis between species composition and predicted resistance genes profiles were conducted using the corrplot package version 0.92^108^ and visualised into graphical network using tidygraph version 1.2.3^109^, igraph version 1.4.2^110^, and ggraph version 2.1.0^111^.

### Reporting summary

Further information on research design is available in the Nature Research Reporting Summary linked to this article.

### Supplementary information

Supplementary tables

Supplementary Figures

Reporting Summary

## Data Availability

The raw sequence data used in this study has been uploaded to NCBI under BioProject PRJNA862629.
