# Supplementary material for: Social demographics determinants for resistome and microbiome variation of a multiethnic community in Southern Malaysia
Source: NPJ Biofilms Microbiomes. 2023 Aug 12;9:55. doi: 10.1038/s41522-023-00425-0 (PMC10423249; doi:10.1038/s41522-023-00425-0)
Supplement: Supplementary file 2 — Supplementary Figures [file 41522_2023_425_MOESM2_ESM.pdf]

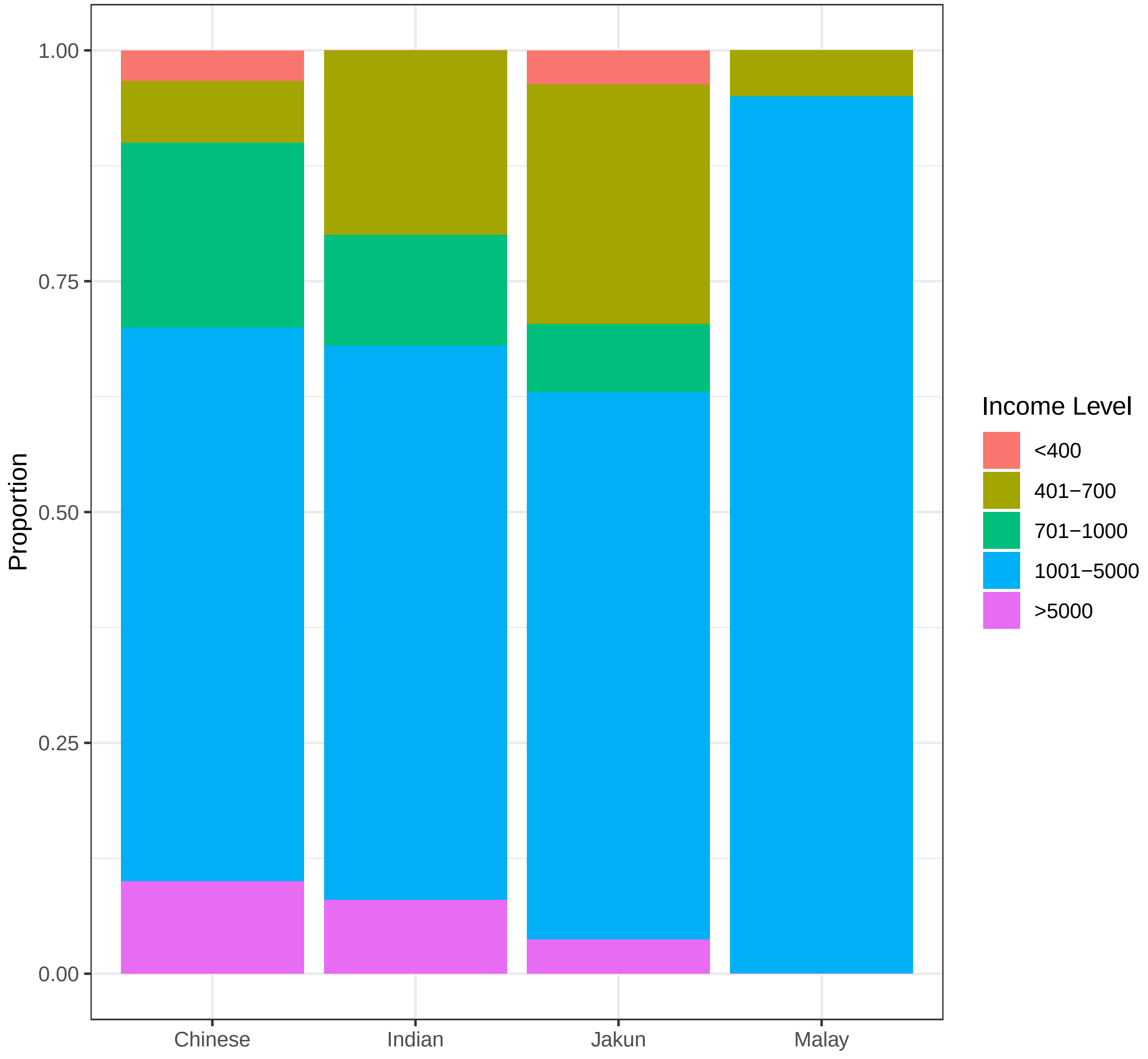

Supplementary Figure 1
Household income distribution of the studied cohort.

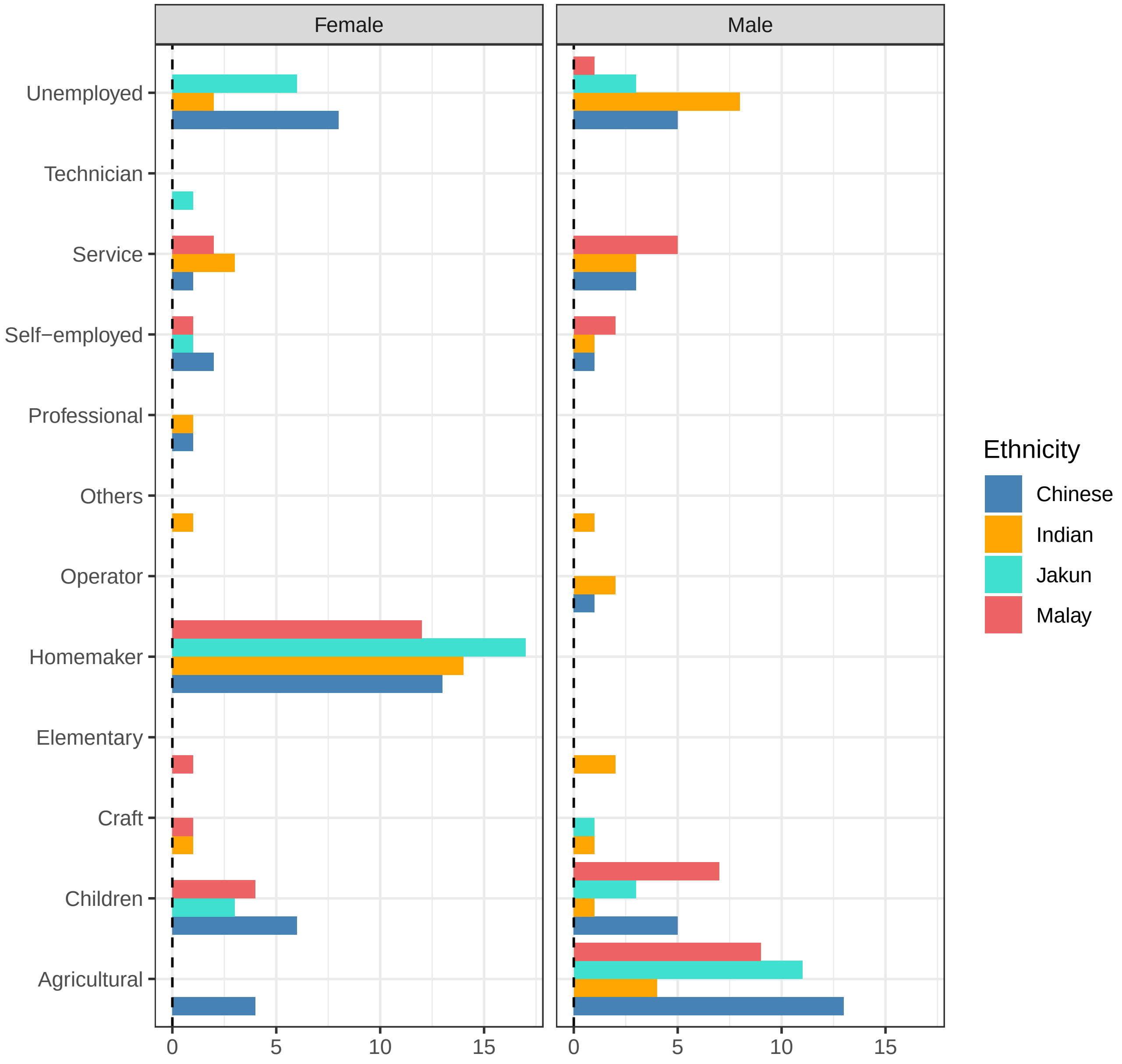

Supplementary Figure 2

Occupation distribution of the studied cohort classified based on sex.

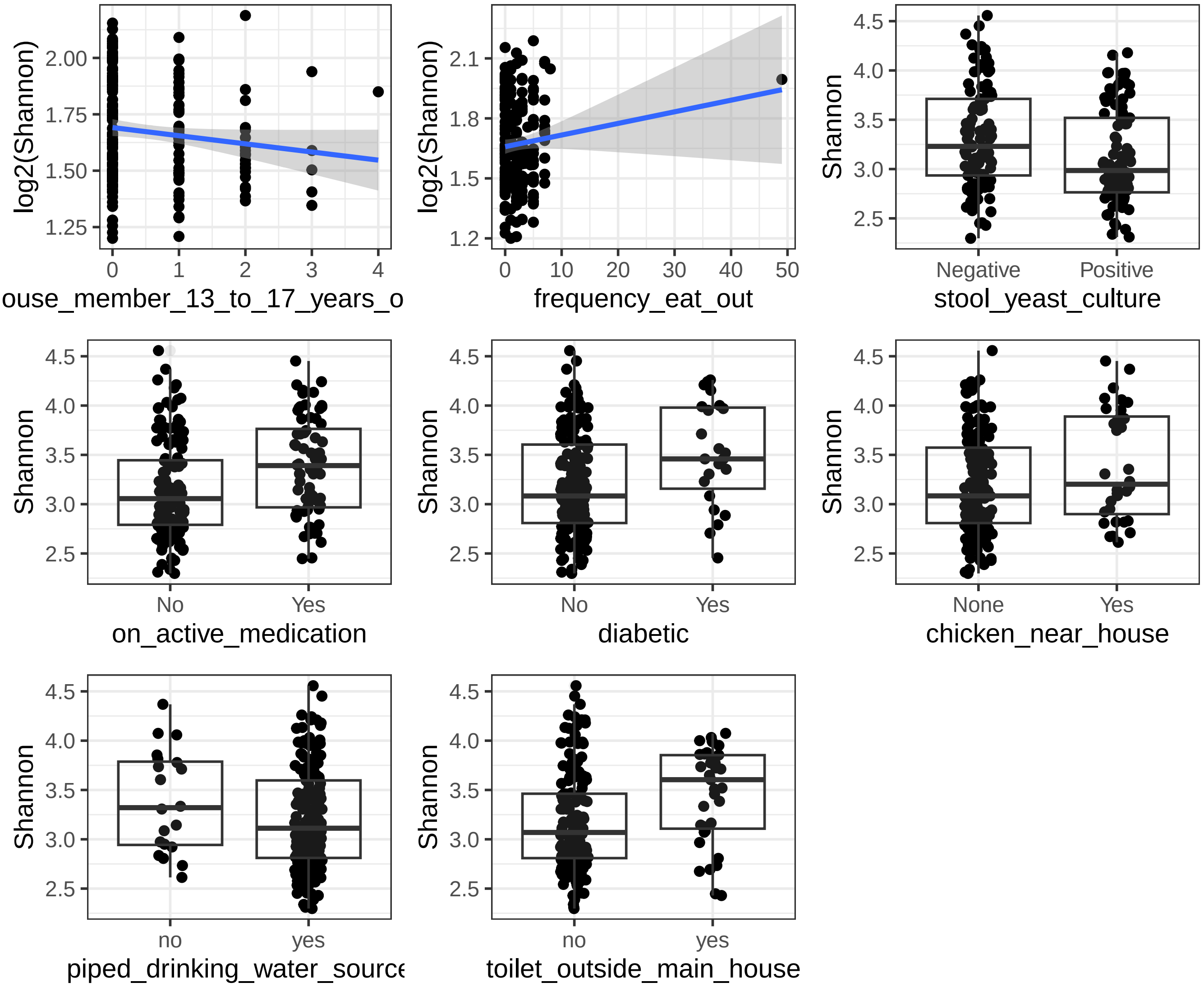

Supplementary Figure 3

Lifestyle and demographic factors significantly associated with the Shannon diversity index of the resistome profile (LRT p<0.1). The boxplot's lower and upper boundaries marked the first (25th percentile) and the third (75th percentile) quartile of the visualised data, while the middle hinge marked the median. The lower and upper whiskers marked the lowest and highest values no smaller/larger than 1.5×interquartile range of the visualised dataset, respectively.

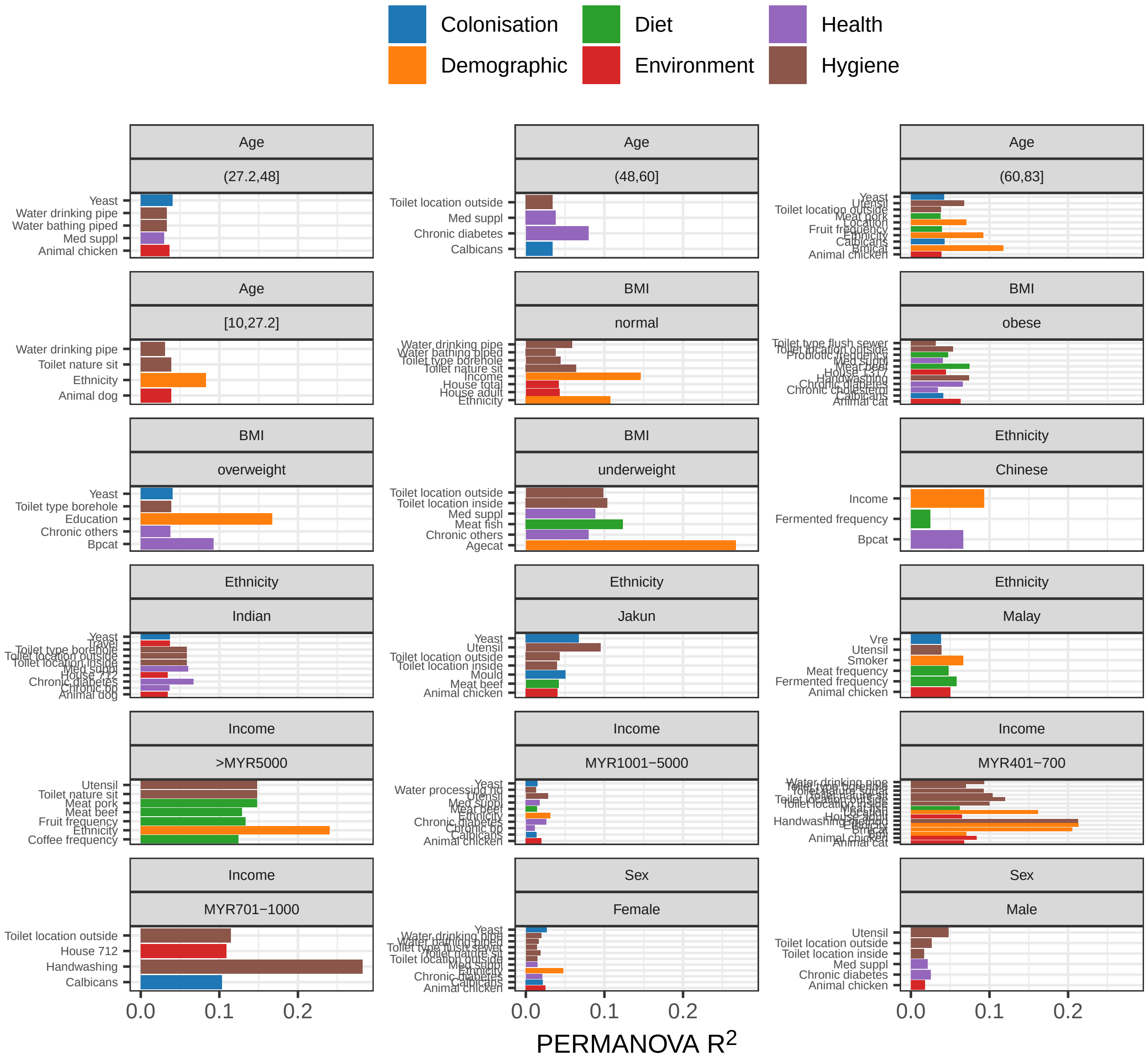

Supplementary Figure 4

Subgroup analysis of lifestyle factors and their association with the gut resistome profile based on adjusted PERMANOVA, stratified based on age, sex, ethnicity, income, and BMI.

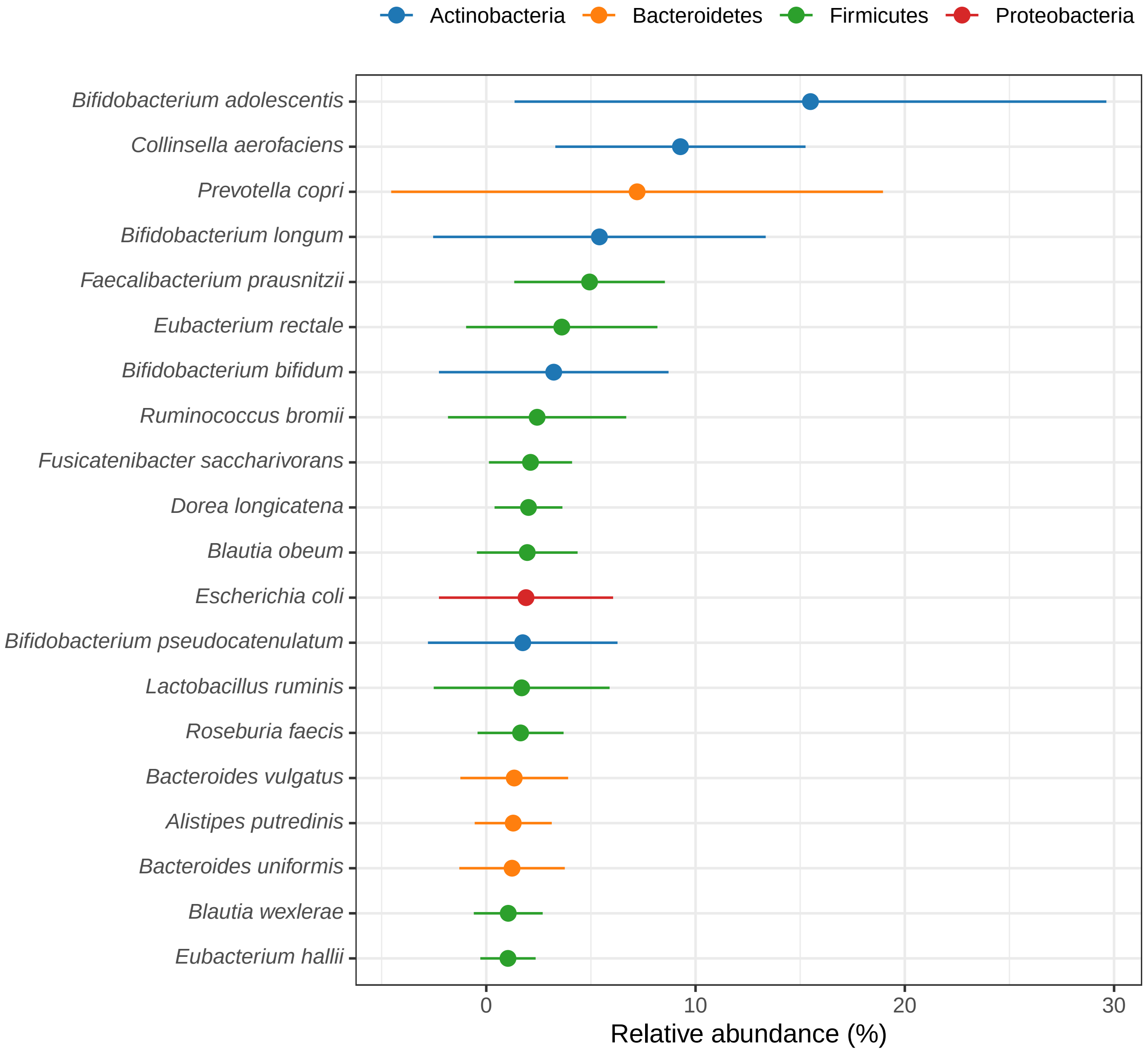

Supplementary Figure 5

Top 20 most abundant microbial species in the stool culture of the Segamat cohort. The point refers to the mean of the visualised dataset, while the error bar refers to 1× standard deviation of the visualised dataset.

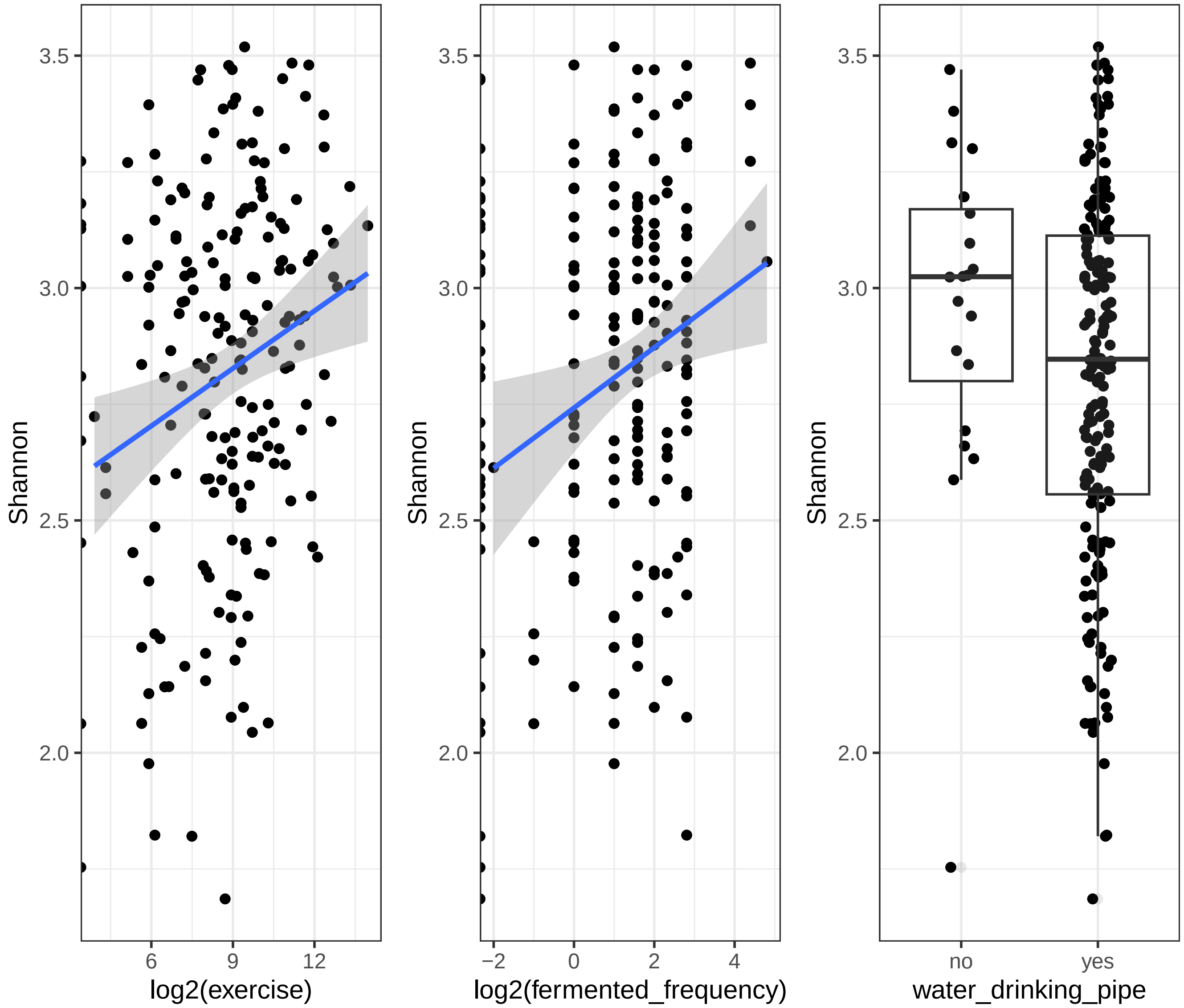

Supplementary Figure 6

Lifestyle and demographic factors significantly associated with the Shannon diversity index of the gut microbiota. The boxplot's lower and upper boundaries marked the first (25th percentile) and the third (75th percentile) quartile of the visualised data, while the middle hinge marked the median. The lower and upper whiskers marked the lowest and highest values no smaller/larger than 1.5×interquartile range of the visualised dataset, respectively.

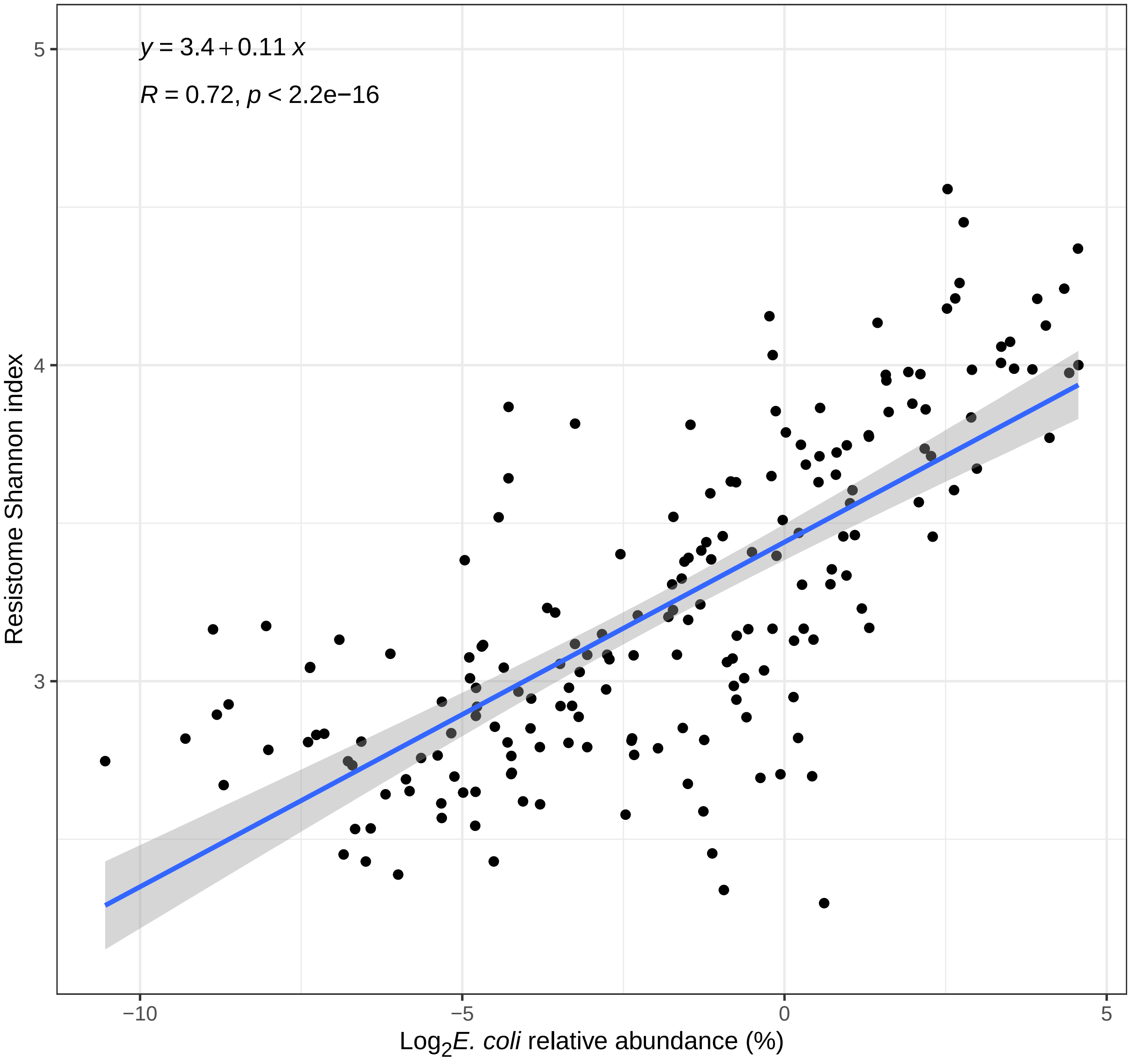

Supplementary Figure 7

Escherichia coli abundance and the resistome Shannon diversity.
