# Supplementary material for: Social demographics determinants for resistome and microbiome variation of a multiethnic community in Southern Malaysia
Source: NPJ Biofilms Microbiomes. 2023 Aug 12;9:55. doi: 10.1038/s41522-023-00425-0 (PMC10423249; doi:10.1038/s41522-023-00425-0)
Supplement: Supplementary file 3 — Reporting Summary [file 41522_2023_425_MOESM3_ESM.pdf]

nature portfolio

Corresponding author(s): Jacky Dwiyanto, Chun Wie Chong

Last updated by author(s): May 31, 2023

Reporting Summary

Nature Portfolio wishes to improve the reproducibility of the work that we publish. This form provides structure for consistency and transparency in reporting. For further information on Nature Portfolio policies, see our Editorial Policies and the Editorial Policy Checklist.

Statistics

For all statistical analyses, confirm that the following items are present in the figure legend, table legend, main text, or Methods section.

n/a	Confirmed	
☐	☒	The exact sample size (*n*) for each experimental group/condition, given as a discrete number and unit of measurement
☐	☒	A statement on whether measurements were taken from distinct samples or whether the same sample was measured repeatedly
☐	☒	The statistical test(s) used AND whether they are one- or two-sided<br>*Only common tests should be described solely by name; describe more complex techniques in the Methods section.*
☐	☒	A description of all covariates tested
☐	☒	A description of any assumptions or corrections, such as tests of normality and adjustment for multiple comparisons
☐	☒	A full description of the statistical parameters including central tendency (e.g. means) or other basic estimates (e.g. regression coefficient) AND variation (e.g. standard deviation) or associated estimates of uncertainty (e.g. confidence intervals)
☒	☐	For null hypothesis testing, the test statistic (e.g. *F*, *t*, *r*) with confidence intervals, effect sizes, degrees of freedom and *P* value noted<br>*Give P values as exact values whenever suitable.*
☒	☐	For Bayesian analysis, information on the choice of priors and Markov chain Monte Carlo settings
☒	☐	For hierarchical and complex designs, identification of the appropriate level for tests and full reporting of outcomes
☒	☐	Estimates of effect sizes (e.g. Cohen's *d*, Pearson's *r*), indicating how they were calculated

Our web collection on statistics for biologists contains articles on many of the points above.

Software and code

Policy information about availability of computer code

Data collection	No software was used to collect the data reported in this manuscript.
Data analysis	Analysis and pipeline used for the processing and data analysis are all freely available as open source programs and their source and version has been described in the manuscript.

For manuscripts utilizing custom algorithms or software that are central to the research but not yet described in published literature, software must be made available to editors and reviewers. We strongly encourage code deposition in a community repository (e.g. GitHub). See the Nature Portfolio guidelines for submitting code & software for further information.

Data

Policy information about availability of data

All manuscripts must include a data availability statement. This statement should provide the following information, where applicable:

- Accession codes, unique identifiers, or web links for publicly available datasets
- A description of any restrictions on data availability
- For clinical datasets or third party data, please ensure that the statement adheres to our policy

The raw sequence data used in this study has been uploaded to NCBI under BioProject PRJNA862629 and will be made publicly available upon the publication of this manuscript.

Research involving human participants, their data, or biological material

Policy information about studies with human participants or human data. See also policy information about sex, gender (identity/presentation), and sexual orientation and race, ethnicity and racism.

Reporting on sex and gender	The term "sex" has been used throughout the manuscript to refer to the biological identity of the human participants.
Reporting on race, ethnicity, or other socially relevant groupings	Ethnicity is used throughout the manuscript to describe the four major ethnic groups residing in our study cohort,namely Chinese, Malay, Indian, and Jakun.
Population characteristics	Our cohort is part of the health and demographic surveillance system (HDSS) under South East Asia Community Observatory, which is based in Segamat, Malaysia. The cohort comprises the four major ethnic groups in Malaysia: Chinese, Indian, Jakun, and Malay, and comprises predominantly of subjects in the younger and older age groups. The lesser prevalence of working age adults was similar to the demographic distribution of the Segamat population.
Recruitment	Participants were recruited under the health demographic and surveillance system (HDSS) SEACO, and all participants provided written informed consent.
Ethics oversight	Monash University Human Research Ethics Committee

Note that full information on the approval of the study protocol must also be provided in the manuscript.

Field-specific reporting

Please select the one below that is the best fit for your research. If you are not sure, read the appropriate sections before making your selection.

☐ Life sciences ☐ Behavioural & social sciences ☒ Ecological, evolutionary & environmental sciences

For a reference copy of the document with all sections, see nature.com/documents/nr-reporting-summary-flat.pdf

Ecological, evolutionary & environmental sciences study design

All studies must disclose on these points even when the disclosure is negative.

Study description	Human stool microbiome characterisation and association with lifestyle and demographic variables obtained through questionnaire data
Research sample	Human community participants and their stool sample.
Sampling strategy	Convenient sampling to obtain as many participants as possible within the available research funding. Sampling strategy was employed to obtain relatively equal portion of each major ethnicity in the cohort so as to ensure observed outcome is not ethnic-driven an is an accurate representation of the whole population regardless of demographic variation.
Data collection	Data collectors were trained by SEACO HDSS. SEACO predetermined the candidates for this study by randomly sampling from their community cohort database. Data collectors visited each house to invite the chosen candidates to participate in the study. Participants were also requested for their stool sample, with instructions on proper collection provided and briefed by the data collectors.
Timing and spatial scale	May through June 2018. Sampling was conducted daily on weekday. THe sampling area was limited to the Segamat subdistrict under SEACO's jurisdiction: Chaah, Jabi, and Bekok.
Data exclusions	Children younger than 10 were excluded from the study.
Reproducibility	Not applicable.
Randomization	Candidates for participation was randomly selected from the SEACO community cohort database. THe only factor controlled was the recruitment of relatively equal number of each ethnic groups so as to obtain a representative sample of the multiracial community.
Blinding	The laboratory staff receiving and processing the biological specimen were blinded from the identity of the community participants to avoid any unconscious bias in processing the samples.

Did the study involve field work? ☐ Yes ☒ No

Reporting for specific materials, systems and methods

We require information from authors about some types of materials, experimental systems and methods used in many studies. Here, indicate whether each material, system or method listed is relevant to your study. If you are not sure if a list item applies to your research, read the appropriate section before selecting a response.

Materials & experimental systems

n/a	Involved in the study
☒	☐ Antibodies
☒	☐ Eukaryotic cell lines
☒	☐ Palaeontology and archaeology
☒	☐ Animals and other organisms
☒	☐ Clinical data
☒	☐ Dual use research of concern
☒	☐ Plants

Methods

n/a	Involved in the study
☒	☐ ChIP-seq
☒	☐ Flow cytometry
☒	☐ MRI-based neuroimaging
